# 1,2,3-Tri-*O*-acetyl-5-de­oxy-d-ribofuran­ose

**DOI:** 10.1107/S160053681004482X

**Published:** 2010-11-10

**Authors:** Wen-Jian Tang, Zhi-Cai Lin, Min-Fang Tang, Jun Li

**Affiliations:** a School of Pharmacy, Anhui Medical University, Hefei 230032, People’s Republic of China

## Abstract

The title compound, C_11_H_16_O_7_, was obtained from the breakage reaction of the glycosidic bond of 5′-de­oxy-2′,3′-diacetyl­inosine. The ribofuran­ose ring has a C2-*exo*, C3-*endo* twist configuration. No alteration of the relative configuration compared with d-(−)-ribose is observed.

## Related literature

For possible catalytic mechanisms at the anomeric carbon centre in the cleavage of glycosidic linkages, see: Vocadlo *et al.* (2001 ▶). For the synthesis of the title compound from d-ribose, see: Sairam *et al.* (2003 ▶). For a 5-de­oxy-ribofuran­oid active as an anti­tumour drug, see: Shimma *et al.* (2000 ▶).
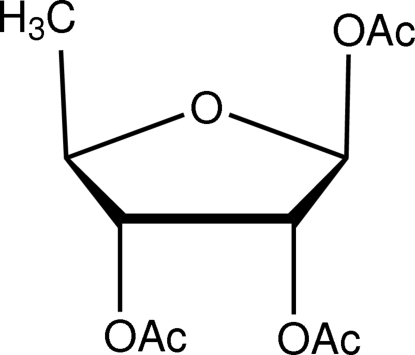


## Experimental

### 

#### Crystal data

C_11_H_16_O_7_
*M* *_r_* = 260.24
Orthorhombic, 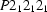

*a* = 7.592 (2) Å
*b* = 8.505 (2) Å
*c* = 20.445 (2) Å
*V* = 1320.1 (5) Å^3^
*Z* = 4
Mo *K*α radiation
μ = 0.11 mm^−1^
*T* = 298 K
0.48 × 0.45 × 0.32 mm

#### Data collection

Siemens SMART 1000 CCD area-detector diffractometer
Absorption correction: multi-scan (*SADABS*; Sheldrick, 1996 ▶) *T*_min_ = 0.949, *T*_max_ = 0.966
5470 measured reflections
1368 independent reflections
848 reflections with *I* > 2σ(*I*)
*R*_int_ = 0.036

#### Refinement

*R*[*F* ^2^ > 2σ(*F* ^2^)] = 0.041
*wR*(*F* ^2^) = 0.133
*S* = 1.04
1368 reflections
168 parameters
H-atom parameters constrained
Δρ_max_ = 0.18 e Å^−3^
Δρ_min_ = −0.13 e Å^−3^

### 

Data collection: *SMART* (Siemens, 1996 ▶); cell refinement: *SAINT* (Siemens, 1996 ▶); data reduction: *SAINT*; program(s) used to solve structure: *SHELXS97* (Sheldrick, 2008 ▶); program(s) used to refine structure: *SHELXL97* (Sheldrick, 2008 ▶); molecular graphics: *SHELXTL* (Sheldrick, 2008 ▶); software used to prepare material for publication: *SHELXL97*.

## Supplementary Material

Crystal structure: contains datablocks I, global. DOI: 10.1107/S160053681004482X/si2303sup1.cif

Structure factors: contains datablocks I. DOI: 10.1107/S160053681004482X/si2303Isup2.hkl

Additional supplementary materials:  crystallographic information; 3D view; checkCIF report

## Figures and Tables

**Fig. 1.**
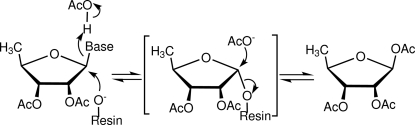
Possible formation mechanisms of the title compound.

**Fig. 2.**
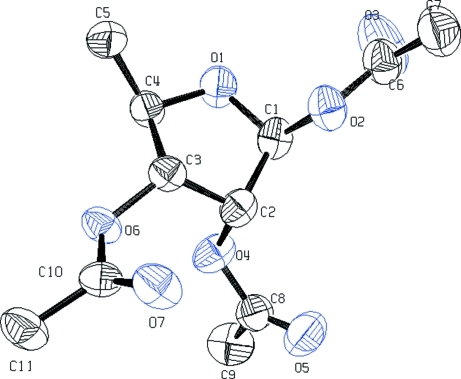
ORTEP drawing of the title compound with atomic numbering scheme and thermal ellipsoids at 30% probability level.

## supplementary crystallographic information

### Comment

During the last decades there has been considerable interest in the chemical synthesis of the nucleoside analogues for their biological evaluation of the anti-tumor activity (Sairam *et al.*, 2003). 1,2,3-*O*-Triacetyl-5-deoxy-D-ribofuranose, as one of the important intermediates, was used to synthesize some anti-cancer drugs such as Doxifluridine, Capecitabine (Shimma *et al.*, 2000), and so on. There were different synthetic routes available in literature for the synthesis of this intermediate. We obtained this compound from inosine as starting material in a linear synthetic route. Possible formation mechanisms of the title compound are shown in Fig. 1. To know the relative stereochemistry of the anomeric position in the ribose, it is therefore necessary to gain the well defined structure of the 1,2,3-*O*-triacetyl-5-deoxy-D-ribofuranose by X-diffraction method (Fig. 2).

We observed that the ribofuranose ring has a C2-*exo*, C3-*endo* twist configuration and the anomeric carbons are always β configuration in the crystal packing. We suppose that the mechanism of the breakage reaction of the glycosidic bond is similar to that of the glycoside hydrolase (Vocadlo *et al.*, 2001). Firstly, the nucleophilic group of the cation resin attacks the anomeric centre of the 5'-deoxy-2',3'-diacetyl-inosine, resulting in the formation of a glycosyl intermediate. Then a nucleophilic acetic anhydride as a base acts the glycosyl intermediate by acetolysis, giving the title product. In another way, the product obtained with sulfuric acid as catalyst is a α/β anomeric mixture and the yield is much lower. This difference may be because the intermediate produced using strong acid is a carbocation and the furan ring may be decomposed to some byproducts.

### Experimental

The title compound was prepared from the reaction of the breakage of the glycosidic bond of 5'-Deoxy-2',3'-diacetyl-inosine, which was gained from inosine by halogenation, hydrogenization and acetylation in turn. 5'-Deoxy-2',3'-diacetyl-inosine (6.72 g, 20 mmol) and cation-exchange resin (6 g) were added to a solution of acetic anhydride/acetic acid (60 ml, 9: 1), was heated to 358 K and reacted under stirring for 8 h. The reacting mixture was filtered and the filtrate was concentrated *in vacuo*. The residue was resolved in ethyl acetate, then the precipitate was filtered and the filtrate was washed by the saturated solution of NaHCO_3_. The organic layer was dried with anhydrous Na_2_SO_4_, filtered, and concentrated *in vacuo*. The residue was recrystallized from methanol/water. The purified title compound was subsequently dissolved in methanol and added water to the solution until it turned cloudy. Upon standing at room temperature, a colorless block appeared and was separated from the solvent by decantation.

### Refinement

All H atoms were positioned geometrically and refined using a riding model, with C—H distances of 0.98 Å (methyl), with *U*_iso_(H) = 1.2*U*_eq_(C) and 1.5*U*_eq_(*C*-methyl).

In the absence of significant anomalous scattering effects, Friedel pairs were averaged.

### Crystal data

**Table:**

C_11_H_16_O_7_	*D*_x_ = 1.309 Mg m^−^^3^
*M**_r_* = 260.24	Mo *K*α radiation, λ = 0.71073 Å
Orthorhombic, *P*2_1_2_1_2_1_	Cell parameters from 1390 reflections
*a* = 7.592 (2) Å	θ = 2.6–21.6°
*b* = 8.505 (2) Å	µ = 0.11 mm^−^^1^
*c* = 20.445 (2) Å	*T* = 298 K
*V* = 1320.1 (5) Å^3^	Prism, colourless
*Z* = 4	0.48 × 0.45 × 0.32 mm
*F*(000) = 552	

### Data collection

**Table:**

Siemens SMART 1000 CCD area-detector diffractometer	1368 independent reflections
Radiation source: fine-focus sealed tube	848 reflections with *I* > 2σ(*I*)
graphite	*R*_int_ = 0.036
φ and ω scans	θ_max_ = 25.0°, θ_min_ = 2.0°
Absorption correction: multi-scan (*SADABS*; Sheldrick, 1996)	*h* = −9→8
*T*_min_ = 0.949, *T*_max_ = 0.966	*k* = −10→9
5470 measured reflections	*l* = −24→11

### Refinement

**Table:**

Refinement on *F*^2^	Secondary atom site location: difference Fourier map
Least-squares matrix: full	Hydrogen site location: inferred from neighbouring sites
*R*[*F*^2^ > 2σ(*F*^2^)] = 0.041	H-atom parameters constrained
*wR*(*F*^2^) = 0.133	*w* = 1/[σ^2^(*F*_o_^2^) + (0.0609*P*)^2^ + 0.2525*P*] where *P* = (*F*_o_^2^ + 2*F*_c_^2^)/3
*S* = 1.04	(Δ/σ)_max_ < 0.001
1368 reflections	Δρ_max_ = 0.18 e Å^−^^3^
168 parameters	Δρ_min_ = −0.13 e Å^−^^3^
0 restraints	Extinction correction: *SHELXL97* (Sheldrick, 2008), Fc^*^=kFc[1+0.001xFc^2^λ^3^/sin(2θ)]^-1/4^
Primary atom site location: structure-invariant direct methods	Extinction coefficient: 0.022 (4)

### Special details

**Table:**

Geometry. All esds (except the esd in the dihedral angle between two l.s. planes) are estimated using the full covariance matrix. The cell esds are taken into account individually in the estimation of esds in distances, angles and torsion angles; correlations between esds in cell parameters are only used when they are defined by crystal symmetry. An approximate (isotropic) treatment of cell esds is used for estimating esds involving l.s. planes.
Refinement. Refinement of *F*^2^ against ALL reflections. The weighted *R*-factor *wR* and goodness of fit *S* are based on *F*^2^, conventional *R*-factors *R* are based on *F*, with *F* set to zero for negative *F*^2^. The threshold expression of *F*^2^ > σ(*F*^2^) is used only for calculating *R*-factors(gt) *etc*. and is not relevant to the choice of reflections for refinement. *R*-factors based on *F*^2^ are statistically about twice as large as those based on *F*, and *R*- factors based on ALL data will be even larger.

### Fractional atomic coordinates and isotropic or equivalent isotropic displacement parameters (Å^2^)

**Table:**

	*x*	*y*	*z*	*U*_iso_*/*U*_eq_	
O1	0.1176 (4)	0.2987 (4)	0.07061 (14)	0.0875 (10)	
O2	0.2931 (4)	0.3413 (4)	0.16197 (14)	0.0848 (10)	
O3	0.4083 (8)	0.5348 (6)	0.1051 (2)	0.162 (2)	
O4	0.2959 (4)	−0.0158 (4)	0.07017 (13)	0.0873 (11)	
O5	0.5372 (5)	−0.0822 (5)	0.1230 (2)	0.1261 (16)	
O6	−0.0276 (4)	−0.0744 (3)	0.12588 (12)	0.0711 (9)	
O7	0.0619 (5)	−0.1341 (4)	0.22641 (13)	0.0952 (12)	
C1	0.2710 (6)	0.2524 (6)	0.1032 (2)	0.0750 (13)	
H1	0.3742	0.2633	0.0748	0.090*	
C2	0.2469 (6)	0.0844 (6)	0.12401 (18)	0.0695 (12)	
H2	0.3104	0.0591	0.1644	0.083*	
C3	0.0506 (6)	0.0772 (5)	0.13214 (17)	0.0628 (11)	
H3	0.0192	0.1214	0.1748	0.075*	
C4	−0.0187 (6)	0.1839 (5)	0.07907 (19)	0.0678 (11)	
H4	−0.0317	0.1235	0.0385	0.081*	
C5	−0.1893 (6)	0.2655 (5)	0.0940 (2)	0.0917 (16)	
H5A	−0.1762	0.3274	0.1330	0.138*	
H5B	−0.2802	0.1886	0.1005	0.138*	
H5C	−0.2206	0.3327	0.0581	0.138*	
C6	0.3685 (7)	0.4813 (7)	0.1569 (3)	0.0917 (15)	
C7	0.3865 (8)	0.5618 (7)	0.2211 (3)	0.1153 (19)	
H7A	0.4613	0.5007	0.2492	0.173*	
H7B	0.2725	0.5726	0.2409	0.173*	
H7C	0.4374	0.6639	0.2146	0.173*	
C8	0.4508 (7)	−0.0888 (6)	0.0750 (2)	0.0784 (13)	
C9	0.4968 (8)	−0.1728 (7)	0.0142 (2)	0.111 (2)	
H9A	0.5860	−0.1152	−0.0088	0.166*	
H9B	0.3940	−0.1822	−0.0128	0.166*	
H9C	0.5403	−0.2757	0.0248	0.166*	
C10	−0.0159 (6)	−0.1698 (5)	0.1781 (2)	0.0681 (11)	
C11	−0.1095 (8)	−0.3204 (6)	0.1666 (2)	0.0968 (16)	
H11A	−0.0379	−0.3877	0.1399	0.145*	
H11B	−0.2191	−0.3001	0.1448	0.145*	
H11C	−0.1320	−0.3709	0.2078	0.145*	

### Atomic displacement parameters (Å^2^)

**Table:**

	*U*^11^	*U*^22^	*U*^33^	*U*^12^	*U*^13^	*U*^23^
O1	0.090 (2)	0.087 (2)	0.086 (2)	−0.018 (2)	−0.0159 (18)	0.0309 (18)
O2	0.095 (2)	0.089 (2)	0.0703 (19)	−0.023 (2)	−0.0001 (17)	0.0068 (19)
O3	0.232 (6)	0.137 (4)	0.116 (3)	−0.093 (4)	0.011 (4)	0.022 (3)
O4	0.087 (2)	0.115 (3)	0.0599 (17)	0.025 (2)	−0.0058 (16)	−0.0044 (18)
O5	0.099 (3)	0.135 (4)	0.145 (3)	0.029 (3)	−0.043 (3)	−0.031 (3)
O6	0.098 (2)	0.0548 (17)	0.0604 (16)	−0.0068 (19)	−0.0069 (15)	0.0085 (14)
O7	0.148 (3)	0.079 (2)	0.0585 (17)	−0.009 (2)	−0.0165 (19)	0.0096 (16)
C1	0.070 (3)	0.095 (4)	0.060 (2)	−0.010 (3)	0.000 (2)	0.004 (2)
C2	0.073 (3)	0.088 (3)	0.048 (2)	0.006 (3)	−0.006 (2)	0.001 (2)
C3	0.077 (3)	0.062 (3)	0.050 (2)	0.001 (3)	−0.0017 (19)	0.010 (2)
C4	0.077 (3)	0.064 (2)	0.062 (2)	−0.008 (3)	−0.012 (2)	0.010 (2)
C5	0.081 (3)	0.074 (3)	0.120 (4)	0.003 (3)	−0.013 (3)	0.016 (3)
C6	0.100 (4)	0.086 (4)	0.089 (4)	−0.024 (3)	−0.009 (3)	0.015 (3)
C7	0.132 (5)	0.101 (4)	0.112 (4)	−0.019 (4)	−0.028 (4)	−0.003 (4)
C8	0.073 (3)	0.076 (3)	0.086 (3)	−0.002 (3)	0.005 (3)	0.007 (3)
C9	0.118 (4)	0.113 (4)	0.102 (4)	0.007 (4)	0.030 (3)	0.008 (4)
C10	0.086 (3)	0.062 (3)	0.057 (2)	0.009 (3)	0.006 (2)	0.011 (2)
C11	0.135 (4)	0.067 (3)	0.088 (3)	−0.015 (3)	−0.014 (3)	0.018 (3)

### Geometric parameters (Å, °)

**Table:**

O1—C1	1.399 (5)	C4—C5	1.501 (6)
O1—C4	1.433 (5)	C4—H4	0.9800
O2—C6	1.325 (6)	C5—H5A	0.9600
O2—C1	1.429 (5)	C5—H5B	0.9600
O3—C6	1.192 (6)	C5—H5C	0.9600
O4—C8	1.333 (6)	C6—C7	1.485 (7)
O4—C2	1.441 (5)	C7—H7A	0.9600
O5—C8	1.183 (6)	C7—H7B	0.9600
O6—C10	1.344 (4)	C7—H7C	0.9600
O6—C3	1.425 (5)	C8—C9	1.475 (6)
O7—C10	1.190 (5)	C9—H9A	0.9600
C1—C2	1.502 (6)	C9—H9B	0.9600
C1—H1	0.9800	C9—H9C	0.9600
C2—C3	1.500 (6)	C10—C11	1.484 (6)
C2—H2	0.9800	C11—H11A	0.9600
C3—C4	1.509 (5)	C11—H11B	0.9600
C3—H3	0.9800	C11—H11C	0.9600
			
C1—O1—C4	110.6 (3)	C4—C5—H5C	109.5
C6—O2—C1	117.4 (4)	H5A—C5—H5C	109.5
C8—O4—C2	116.5 (3)	H5B—C5—H5C	109.5
C10—O6—C3	116.6 (3)	O3—C6—O2	121.4 (5)
O1—C1—O2	110.4 (4)	O3—C6—C7	125.8 (5)
O1—C1—C2	107.5 (4)	O2—C6—C7	112.7 (5)
O2—C1—C2	106.3 (3)	C6—C7—H7A	109.5
O1—C1—H1	110.8	C6—C7—H7B	109.5
O2—C1—H1	110.8	H7A—C7—H7B	109.5
C2—C1—H1	110.8	C6—C7—H7C	109.5
O4—C2—C1	108.4 (3)	H7A—C7—H7C	109.5
O4—C2—C3	108.5 (4)	H7B—C7—H7C	109.5
C1—C2—C3	101.0 (4)	O5—C8—O4	121.9 (5)
O4—C2—H2	112.7	O5—C8—C9	126.3 (5)
C1—C2—H2	112.7	O4—C8—C9	111.8 (5)
C3—C2—H2	112.7	C8—C9—H9A	109.5
O6—C3—C2	116.1 (4)	C8—C9—H9B	109.5
O6—C3—C4	109.5 (3)	H9A—C9—H9B	109.5
C2—C3—C4	104.0 (3)	C8—C9—H9C	109.5
O6—C3—H3	109.0	H9A—C9—H9C	109.5
C2—C3—H3	109.0	H9B—C9—H9C	109.5
C4—C3—H3	109.0	O7—C10—O6	122.6 (4)
O1—C4—C5	109.4 (4)	O7—C10—C11	126.1 (4)
O1—C4—C3	104.1 (3)	O6—C10—C11	111.4 (4)
C5—C4—C3	115.6 (4)	C10—C11—H11A	109.5
O1—C4—H4	109.1	C10—C11—H11B	109.5
C5—C4—H4	109.1	H11A—C11—H11B	109.5
C3—C4—H4	109.1	C10—C11—H11C	109.5
C4—C5—H5A	109.5	H11A—C11—H11C	109.5
C4—C5—H5B	109.5	H11B—C11—H11C	109.5
H5A—C5—H5B	109.5		
